# Outcomes of Curative Esophagectomy in Octogenarians vs. Non-Octogenarians with Esophageal Cancer: A Systematic Review and Meta-Analysis

**DOI:** 10.3390/geriatrics11030067

**Published:** 2026-06-05

**Authors:** Liyang Xiao, Kai Siang Chan, Isaac Chan, Aung Myint Oo

**Affiliations:** 1 Lee Kong Chian School of Medicine, Nanyang Technological University, Singapore 308232, Singapore; m210137@e.ntu.edu.sg (L.X.); ichan008@e.ntu.edu.sg (I.C.); 2 Upper Gastrointestinal, Bariatric and Metabolic Surgery, Department of General Surgery, Tan Tock Seng Hospital, Singapore 308433, Singapore; 3 Yong Loo Lin School of Medicine, National University of Singapore, Singapore 117597, Singapore

**Keywords:** esophagectomy, esophageal cancer, octogenarians, geriatric oncology

## Abstract

**Background**: Esophageal cancer (EC) poses a growing global challenge in the context of an ageing population. Evidence on the role of curative esophagectomy in octogenarians is limited. This study aims to compare the long-term survival and post-operative mortality and morbidity in octogenarians undergoing curative esophagectomy for EC with those in non-octogenarians. **Methods**: A systematic search was performed on PubMed, Embase, Web of Science and Cochrane Library up to Jan 2026. The inclusion criteria were studies that compared outcomes of esophagectomy for EC between octogenarians and non-octogenarians. Exclusion criteria were single-arm studies and studies using different age cut-offs. **Results**: There were 18 studies with 73,776 patients (octogenarians *n* = 6234 and non-octogenarians patients *n* = 67,542), with smaller subsets of studies being analysed for individual outcomes. The overall incidence of open esophagectomy and minimally invasive esophagectomy (MIE) were 78.4% (*n* = 459/585) and 21.2% (*n* = 124/585), respectively, in octogenarians, and 69.8% (*n* = 3270/4688) and 29.4% (*n* = 1380/4688), respectively, in non-octogenarians. R0 resection was achieved in 85.2% (*n* = 1759/2064) of octogenarians and 91.9% (*n* = 30,764/33,480) of non-octogenarians. Pooled OS was inferior in the octogenarian group compared to the non-octogenarian group (*n* = 35,441, HR 2.29, 95% CI: 1.38–3.79). Pooled in-hospital mortality, 30-day mortality and 90-day mortality were higher in octogenarians. In terms of post-operative complications, pooled analysis demonstrated a higher overall complication rate in the octogenarian group (*n* = 6515, OR 1.40, 95% CI: 1.11–1.78), while rates of anastomosis leakage, chylothorax, respiratory complication, surgical site infection and recurrent laryngeal nerve injury were comparable between the two groups. **Conclusions**: Curative esophagectomy for EC is associated with worse overall survival, mortality and overall post-operative complication rate in octogenarians than non-octogenarians. Further research on the role of MIE in octogenarians should be conducted.

## 1. Introduction

Esophageal cancer (EC) is the eleventh most common cancer and the seventh cause of cancer-related mortality in the world [1]. It confers a poor prognosis, with an overall 5-year survival rate of around 20% [2] and a mortality-to-incidence ratio of 0.86 in males and 0.85 in females globally [3]. Aggressive tumour biology, late presentation and limited treatment options pose tremendous challenges in managing the global burden of EC.

Esophagectomy remains one of the gold standard curative treatments for locally advanced resectable EC [4]. However, esophagectomy is a highly morbid surgery with a risk of peri-operative morbidity and mortality. Studies have reported approximate incidences of anastomotic leakage (10–15%), chylothorax (3–5%), repeat intervention or repeat surgery (13–17%), and post-operative mortality of around 2–4% [5,6]. Post-operative outcomes may be optimised through the implementation of minimally invasive esophagectomy (MIE), which was shown in the MIRO trial to lower post-operative morbidity, and the incorporation of neoadjuvant chemoradiotherapy, as supported by the CROSS trial, which demonstrated superior overall survival [7,8]. Recently, landmark trials such as KEYNOTE-590, CheckMate-577 and ORIENT-15 have shown immunotherapy to be a promising adjunct for improving oncological outcomes in EC [9,10,11].

Furthermore, there is a change in the epidemiology of EC. Between 1991 and 2021, the burden of EC shifted towards older adults, particularly in males, who remain disproportionately affected by the disease. The incidence of EC in males aged 80–84 years and 85–89 years increased by 18% and 41%, respectively [12]. Ageing is associated with physiological decline, increased co-morbidity, frailty and sarcopenia. A meta-analysis by Han et al. in 2019 of 4969 patients demonstrated that octogenarians (aged ≥80 years) had higher in-hospital mortality and worse overall survival (OS) compared to non-octogenarians with EC who underwent curative esophagectomy [13]. Nevertheless, some studies on hepatocellular carcinoma and colorectal cancer have established similar oncological outcomes in carefully selected octogenarians [14,15]. Moreover, recent guidelines have incorporated intensive pre-operative respiratory rehabilitation and nutritional support into standard peri-operative care for esophagectomy [16]. There is also an increasing number of studies supporting the benefits of MIE in octogenarian patients [17,18].

Considering the greater co-morbidity load and physiological decline associated with ageing, it remains important to evaluate whether octogenarians experience similar benefits from esophagectomy as their younger counterparts, given the procedure’s inherent morbidity. As it is impossible to randomise patients based on age, given that age is our exposure variable, available evidence is derived predominantly from retrospective observational studies. This underscores the need for an updated review of the current evidence. Our primary outcome was to compare the long-term survival between octogenarians and non-octogenarians. Our secondary outcomes were to compare the short-term post-operative outcomes, including post-operative complications, in-hospital mortality, 30-day mortality and 90-day mortality following esophagectomy.

## 2. Methods

The systematic review and meta-analysis were performed according to the Preferred Reporting Items for Systematic Reviews and Meta-Analysis (PRISMA) guidelines (Supplementary Methods S1). This study was registered at PROSPERO (ID: CRD420251045584). PubMed, Embase, Web of Science and the Cochrane Library were searched from inception to 5 January 2026 using the following search terms: (“esophagectomy” OR “esophageal surgery” OR “esophageal neoplasms” OR “esophageal cancer”) AND (“older adults” OR “octogenarian*” OR “elderly” OR “geriatric”). The detailed search strategies adapted to each database can be found in Supplementary Methods S2. The bibliographies of included studies were further screened for any additional eligible studies.

The inclusion criteria were studies that compared outcomes of esophagectomy between octogenarian and. younger patients with (1) esophageal cancer and (2) who received esophagectomy with curative intent, with or without other systemic therapy. The exclusion criteria were (1) single-arm studies without a comparator group, (2) studies using an age cut-off other than 80 years between the two groups of patients, (3) case reports, case series, conference abstracts and review articles, (4) studies that did not include short-term or long-term post-operative outcomes, and (5) non-English studies. After removal of duplicates, all the abstracts were screened by two independent authors (L.X. and I.C.) based on the above inclusion and exclusion criteria. Full texts of included articles were then scrutinised and selected based on the above criteria. Any discrepancies were resolved through discussion with the senior authors (K.S.C. and A.M.O.).

### 2.1. Data Extraction

Data were collected independently by two authors (L.X. and I.C.). The following variables were collected: basic study characteristics (study design, study period, country, and sample size), patient demographics (age, gender, pre-existing co-morbidities, including hypertension, diabetes, cardiovascular disease and respiratory disease), tumour characteristics (tumour location, lymph node involvement, clinical stage, and histology type), neoadjuvant and/or adjuvant therapy details and details on esophagectomy (surgery approach, esophagectomy type, and resection margin). Our primary outcomes were overall survival (OS) and disease-free survival (DFS). Our secondary outcomes were post-operative complications and short-term mortality. For studies that used propensity score matching (PSM), only the PSM cohort was included in our review.

### 2.2. Assessment of Study Quality

The Newcastle–Ottawa Scale for Cohort Studies was used to assess the bias of non-randomised observation studies (Supplementary Methods S3) [19]. Only studies with a score of >6 were included. Bias was assessed by two independent authors (L.X. and I.C.). Disagreements between authors were resolved through discussion with the senior authors (K.S.C. and A.M.O.).

### 2.3. Statistical Analysis

Categorical variables were described as n (%) and continuous variables were described as mean (SD). Meta-analysis was performed using RevMan 5.4 (Review Manager 5.4, The Nordic Cochrane Centre). In view of the limited number of studies reporting overall survival after esophagectomy in octogenarians compared with non-octogenarians, the hazard ratio (HR) and standard error (SE) were estimated indirectly from the Kaplan–Meier (KM) curves provided in the included studies using the methods described by Parmar et al. [20]. Pooled HRs were calculated through the inverse-variance method using the natural logarithm of HR and SE [21]. For studies that provided KM curves for more than one subgroup of patients aged <80 years, the curves were combined using the algorithm proposed by Liu et al., which reconstructs individual patient data from published survival curves [22]. For studies that reported effect sizes, the effect sizes derived from Cox regression were used for meta-analysis. Heterogeneity was assessed using Cochrane’s Q and quantified by I^2^. A fixed-effect model was used if I^2^ was <50%, and a random-effect model was used when I^2^ was ≥50%. Statistical significance was defined as *p* < 0.05. Post-operative mortality and post-operative complications were weighted and pooled across studies using odds ratios (ORs). Leave-one-out sensitivity analysis was conducted using R (version 4.5.0) and the metafor package (version 4.8-0).

## 3. Results

**Figure 1:**
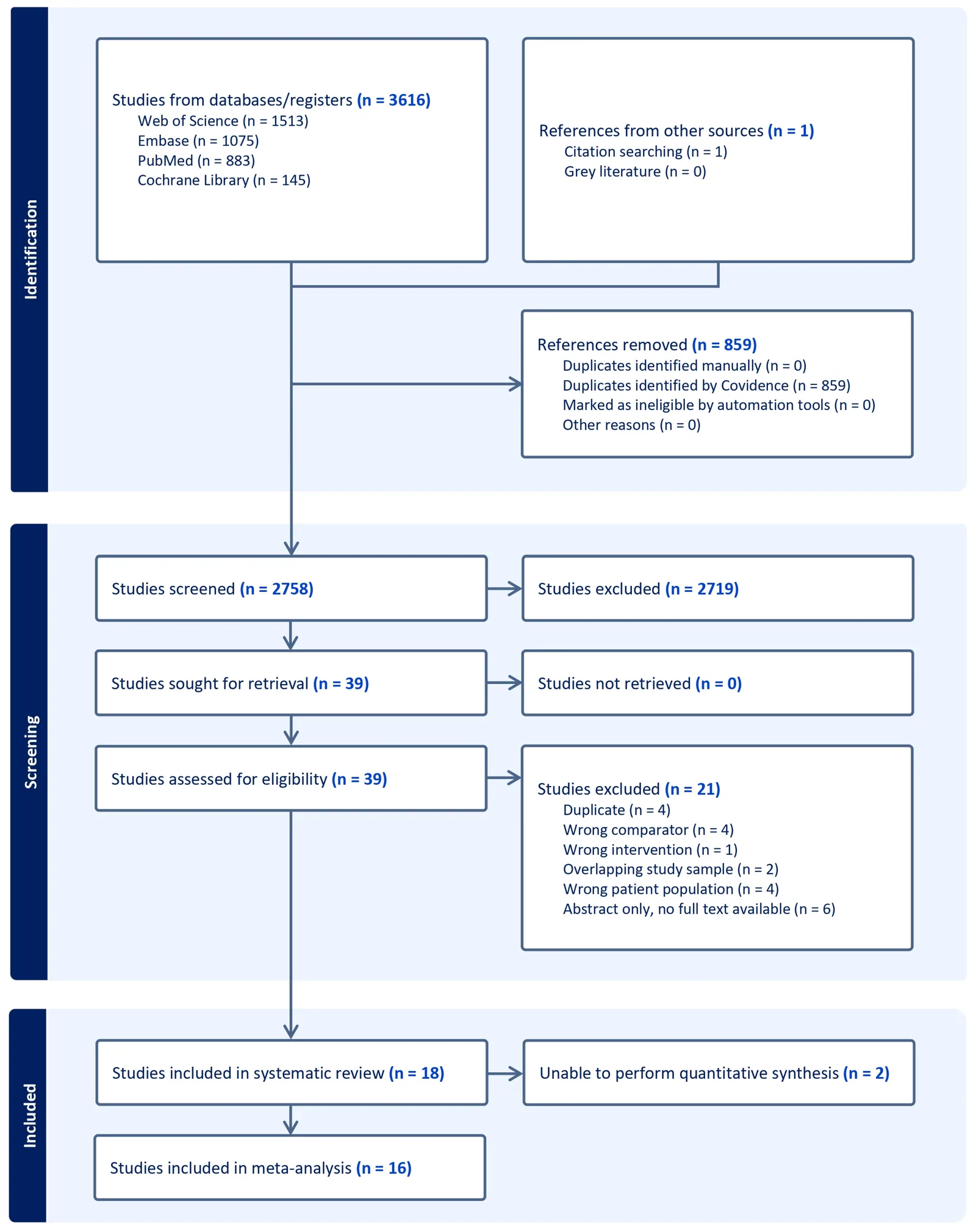
PRISMA (Preferred Reporting Items for Systematic Reviews and Meta-Analyses) flowchart of the study selection process.

There were 3616 studies identified through the search strategy, and one study was obtained from citation searching. After removal of duplicates, 2758 studies were screened based on titles and abstracts, and 39 full-text studies were scrutinised based on the inclusion and exclusion criteria. Eighteen studies were included in the systematic review [23,24,25,26,27,28,29,30,31,32,33,34,35,36,37,38,39,40], and sixteen studies were included in the meta-analysis [23,24,25,26,27,29,30,31,32,34,35,36,37,38,39,40]. The PRISMA flowchart is shown in Figure 1. All eighteen studies are retrospective cohort studies, and one study used PSM [38].

### 3.1. Study Characteristics, Patient Demographics and Tumour Characteristics

There were 18 studies with a total of 73,776 patients (octogenarians: *n* = 6234 (8.4%), non-octogenarians: *n* = 67,542 (91.6%)), with analyses of individual outcomes being derived from a smaller number of studies. Most of the included studies were from Western populations (*n* = 13 studies, 70,075 patients) [23,24,26,27,28,29,32,34,35,37,38,39,40], and four studies (*n* = 3349 patients) were from East Asia [30,31,33,36]. The mean age was 81.5–83.0 years in the octogenarian group and 62.0–63.8 years in the non-octogenarian group. Clinical demographics of the included patients are summarised in Table 1. There were 74.7% (*n* = 1931/2586) and 59.6% (*n* = 21,785/36,532) of octogenarians and non-octogenarians, respectively, with clinical stage I-II EC. Nine studies (*n* = 5807 patients) reported on the location of EC [23,25,26,30,31,32,35,36,37], of which 2.1%, 17.0%, 33.8%, and 29.0% of ECs in the octogenarian group, and 6.2%, 24.6%, 26.8%, and 23.0% of ECs in the non-octogenarian group, occurred in the upper third, middle third, lower third, and gastroesophageal junction. Pooled analysis showed no significant differences in the incidence of clinical stage I-II EC, adenocarcinoma and pathological lymph node involvement between the groups (Table 1). Only one study reported HER2 status [26]. None of the included studies reported on the use of prehabilitation. Detailed characteristics of the individual included studies are summarised in Supplementary Tables S1–S4.

### 3.2. Neoadjuvant Therapy Regimen

There were 13.9% (*n* = 98/705) and 38.2% (*n* = 2353/6153) of octogenarians and non-octogenarians, respectively, who received neoadjuvant chemoradiotherapy, with more non-octogenarians receiving neoadjuvant chemoradiotherapy (*n* = 12 studies, 6858 patients; OR 0.40; 95% CI: 0.25–0.64; I^2^ = 53%; *p* = 0.0001) [23,25,26,29,30,32,33,34,35,36,39,40]. There were five studies that reported the regimen of neoadjuvant chemoradiotherapy in the two groups [25,30,36,37,39]. Some of the chemotherapy regimens included fluorouracil + cisplatin, fluorouracil + cisplatin + paclitaxel, carboplatin + paclitaxel, and fluorouracil + leucovorin + oxaliplatin + docetaxel. For radiotherapy regimens, 1.5–2.0 Gy for a total of 40–60 Gy was used. There were 19.3% (*n* = 109/565) and 50.7% (*n* = 1847/3647) of octogenarian and non-octogenarians, respectively, who received neoadjuvant chemotherapy alone (*n* = 6 studies) [23,25,26,30,33,35], and 10.8% (*n* = 52/480) and 35.2% (*n* = 909/2585) of octogenarian and non-octogenarians, respectively, who received neoadjuvant radiotherapy alone (*n* = 3 studies) [23,26,33]. No studies reported on the use of immunotherapy.

### 3.3. Intra-Operative Characteristics

Both open esophagectomy (OE) and MIE were performed in all studies except one, which included OE only [23]. Seven studies reported the specific incidence of each approach [23,26,33,34,37,39,40]. There was only one study that included robot-assisted MIE [37]. Pooled analysis showed no significant difference in MIE adoption rates between octogenarians and non-octogenarians (*n* = 7 studies, 5273 patients; OR 0.87; 95% CI: 0.68–1.11; I^2^ = 0%; *p* = 0.27). Of the 12 studies that reported the on details of the types of esophagectomy [23,25,26,29,30,34,35,36,37,38,39,40], the incidence of Ivor-Lewis, McKeown, trans-hiatal and thoracoabdominal esophagectomies was 52.8% (*n* = 287/544), 4.2% (*n* = 23/544), 16.2% (*n* = 88/544) and 5.9% (*n* = 32/544), respectively, in the octogenarian group, and 50.3% (*n* = 2464/4903), 18.4% (*n* = 904/4903), 11.8% (*n* = 578/4903) and 9.7% (*n* = 478/4903), respectively, in the non-octogenarian group.

Reconstruction types were reported in three studies [31,36,39]. The distribution of esophago-gastric, esophago-colonic, and esophago-jejunal anastomoses was 89.1% (*n* = 41/46), 10.9% (*n* = 5/46) and 0.0% (*n* = 0/46), respectively, in octogenarians, versus 93.9% (*n* = 1548/1648), 4.9% (*n* = 80/1648), and 1.1% (*n* = 19/1648), respectively, in non-octogenarians. Among the two studies that reported anastomosis techniques [23,39], there were 44.2% (*n* = 23/52) hand-sewn anastomoses and 55.8% (*n* = 29/52) stapled anastomoses in octogenarians, versus 52.7% (*n* = 498/945) and 47.3% (*n* = 447/945), respectively, in non-octogenarians. In terms of resection margins, 85.2% (*n* = 1759/2064) of patients in the octogenarian group and 91.9% (*n* = 30,764/33,480) in the non-octogenarian group had R0 resection, and the difference was statistically significant (*n* = 8 studies, 35,544 patients; OR 0.51; 95% CI: 0.45–0.58; I^2^ = 10%; *p* < 0.00001) [23,24,29,30,34,35,36,37]. Average estimated blood loss was reported in three studies [29,32,34], with mean values ranging from 170.3 to 1050.0 mL in octogenarians and from 207.3 to 482 mL in non-octogenarians.

### 3.4. Adjuvant Therapy

Only one study reported the use of adjuvant therapy [26], with the incidence being 1.9% (*n* = 1/52) in octogenarians versus 13.1% (*n* = 142/1083) in non-octogenarians. The specific adjuvant therapy regimen was not reported.

### 3.5. Long-Term Survival

**Figure 2:**
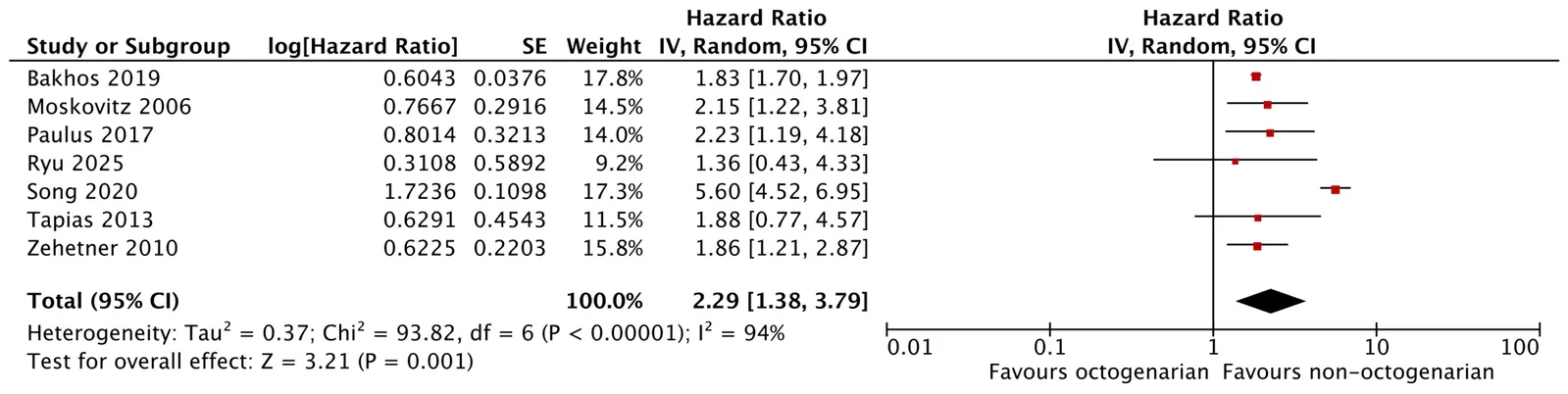
Comparison of overall survival between octogenarians versus non-octogenarians in the included studies [24,32,35,36,37,39,40].

The median OS ranged from 10.0 to 23.0 months in the octogenarian group (*n* = 6 studies, 2069 patients) [23,24,25,32,37,40] and from 29.3 to 62.0 months in the non-octogenarian group (*n* = 3 studies, 31,959 patients) [24,37,40]. The 5-year OS was 15.8–49.2% in the octogenarian group (*n* = 5 studies, 376 patients) [23,30,31,35,39] and 23.9–58.3% in the non-octogenarian group (*n* = 5 studies, 1767 patients) [23,30,31,35,39]. Pooled OS was inferior in octogenarians compared with non-octogenarians (*n* = 7 studies, 35,441 patients; HR 2.29; 95% CI: 1.38–3.79; I^2^ = 94%; *p* < 0.00001) (Figure 2) [24,32,35,36,37,39,40]. Subgroup analyses based on surgical approach and histology types could not be performed due to insufficient stratified data in the included studies. The pooled effect sizes for all post-operative outcomes are summarised in Table 2. None of the included studies reported on disease-free survival, while only two studies reported on cancer-specific survival [39,40]. Of these, one study demonstrated significantly worse cancer-specific survival in octogenarians [39], while the other reported no statistically significant difference [40].

### 3.6. Post-Operative Complications

**Figure 3:**
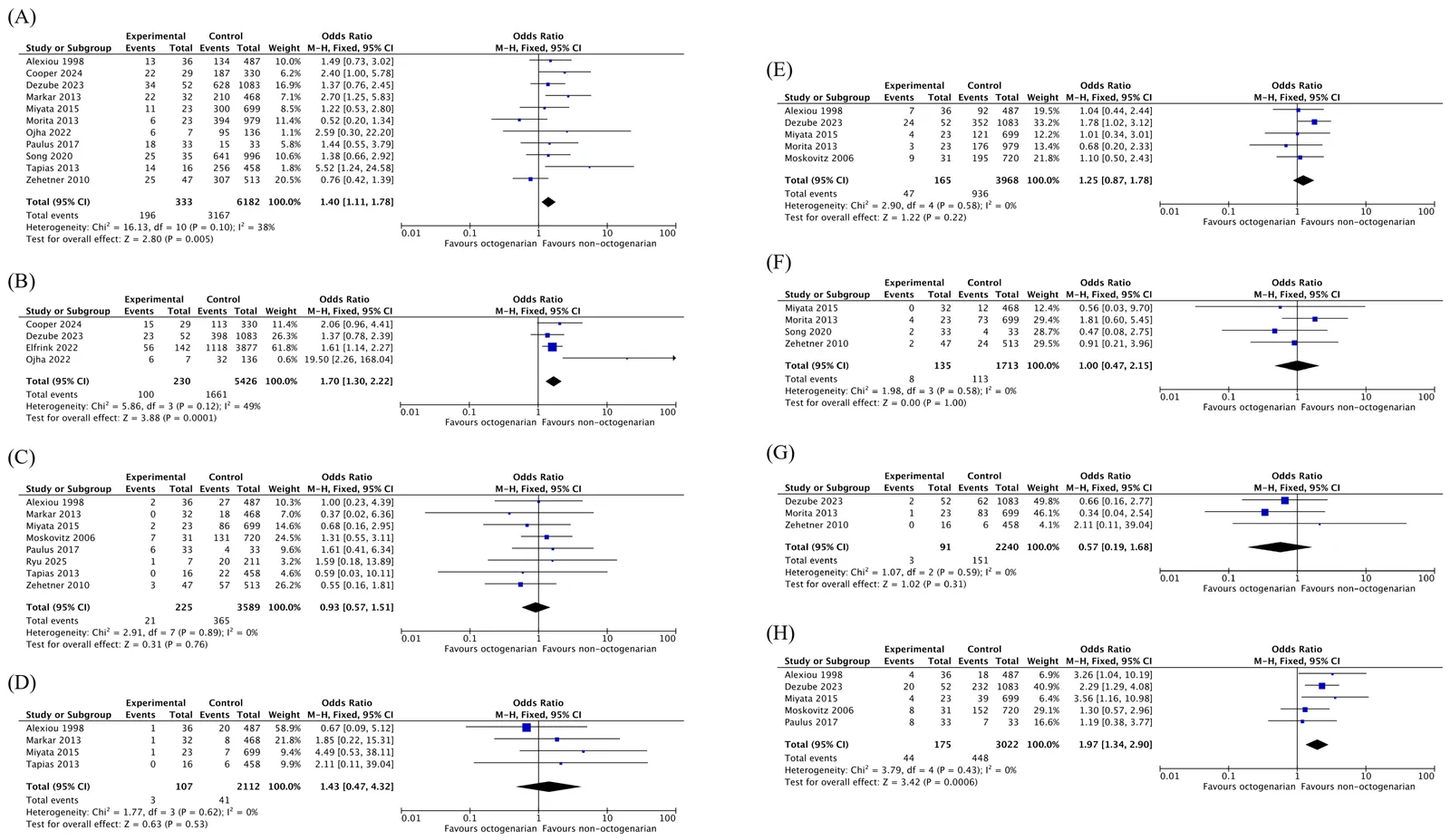
Comparison of (**A**) overall post-operative complication rate [23,25,26,29,30,31,34,35,37,39,40]; (**B**) overall major morbidity [25,26,27,34]; (**C**) anastomotic leakage rate [23,29,30,32,35,36,39,40]; (**D**) chylothorax rate [23,29,30,39]; (**E**) respiratory complications rate [23,26,30,31,32]; (**F**) surgical site infection rate [30,31,37,40]; (**G**) recurrent laryngeal nerve injury rate [26,31,40] and (**H**) cardiovascular complication rate [23,26,30,32,35] between octogenarians and non-octogenarians in the included studies.

The overall incidence of post-operative complications was 58.9% (*n* = 196/333) and 51.2% (*n* = 3167/6182) in octogenarians and non-octogenarians, respectively. Pooled analysis demonstrated a higher overall post-operative complication rate in octogenarians (*n* = 11 studies, 6515 patients; OR 1.40; 95% CI: 1.11–1.78; I^2^ = 38%; *p* = 0.005) (Figure 3A) [23,25,26,29,30,31,34,35,37,39,40]. The overall incidence of major morbidity (Clavien–Dindo ≥ Grade IIIA) was 43.5% (*n* = 100/230) in octogenarians and 30.6% (*n* = 1661/5426) in non-octogenarians, and the difference was statistically significant following pooled analysis (*n* = 4 studies, 5656 patients; OR 1.70; 95% CI: 1.30–2.22; I^2^ = 49%; *p* = 0.0001) (Figure 3B) [25,26,27,34].

The overall incidence of anastomotic leakage, chylothorax, respiratory complications, surgical site infection, recurrent laryngeal nerve injury and cardiovascular complications was 9.3%, 2.8%, 28.5%, 5.9%, 3.3% and 25.1%, respectively, in the octogenarian group, and 10.2%, 1.9%, 23.6%, 6.6%, 6.7% and 14.8%, respectively, in the non-octogenarian group. Pooled analysis showed no statistically significant differences in the rates of anastomotic leakage (*n* = 8 studies, 3814 patients; OR 0.93; 95% CI: 0.57–1.51; I^2^ = 0%; *p* = 0.76) (Figure 3C) [23,29,30,32,35,36,39,40], chylothorax (*n* = 4 studies, 2219 patients; OR 1.43; 95% CI: 0.47–4.32; I^2^ = 0%; *p* = 0.53) (Figure 3D) [23,29,30,39], respiratory complications (*n* = 5 studies, 4133 patients; OR 1.25; 95% CI: 0.87–1.78; I^2^ = 0%; *p* = 0.22) (Figure 3E) [23,26,30,31,32], surgical site infection (*n* = 4 studies, 1848 patients; OR 1.00; 95% CI: 0.47–2.15; I^2^ = 0%; *p* = 1.00) (Figure 3F) [29,30,35,40], and recurrent laryngeal nerve injury (*n* = 3 studies, 2331 patients; OR 0.57; 95% CI: 0.19–1.68; I^2^ = 0%; *p* = 0.31) (Figure 3G) between the two groups [26,30,39]. In terms of cardiovascular complications, pooled analysis demonstrated a worse outcome in the octogenarian group (*n* = 5 studies, 3197 patients; OR 1.97; 95% CI: 1.34–2.90; I^2^ = 0%; *p* = 0.0006) (Figure 3H) [23,26,30,32,35].

**Figure 4:**
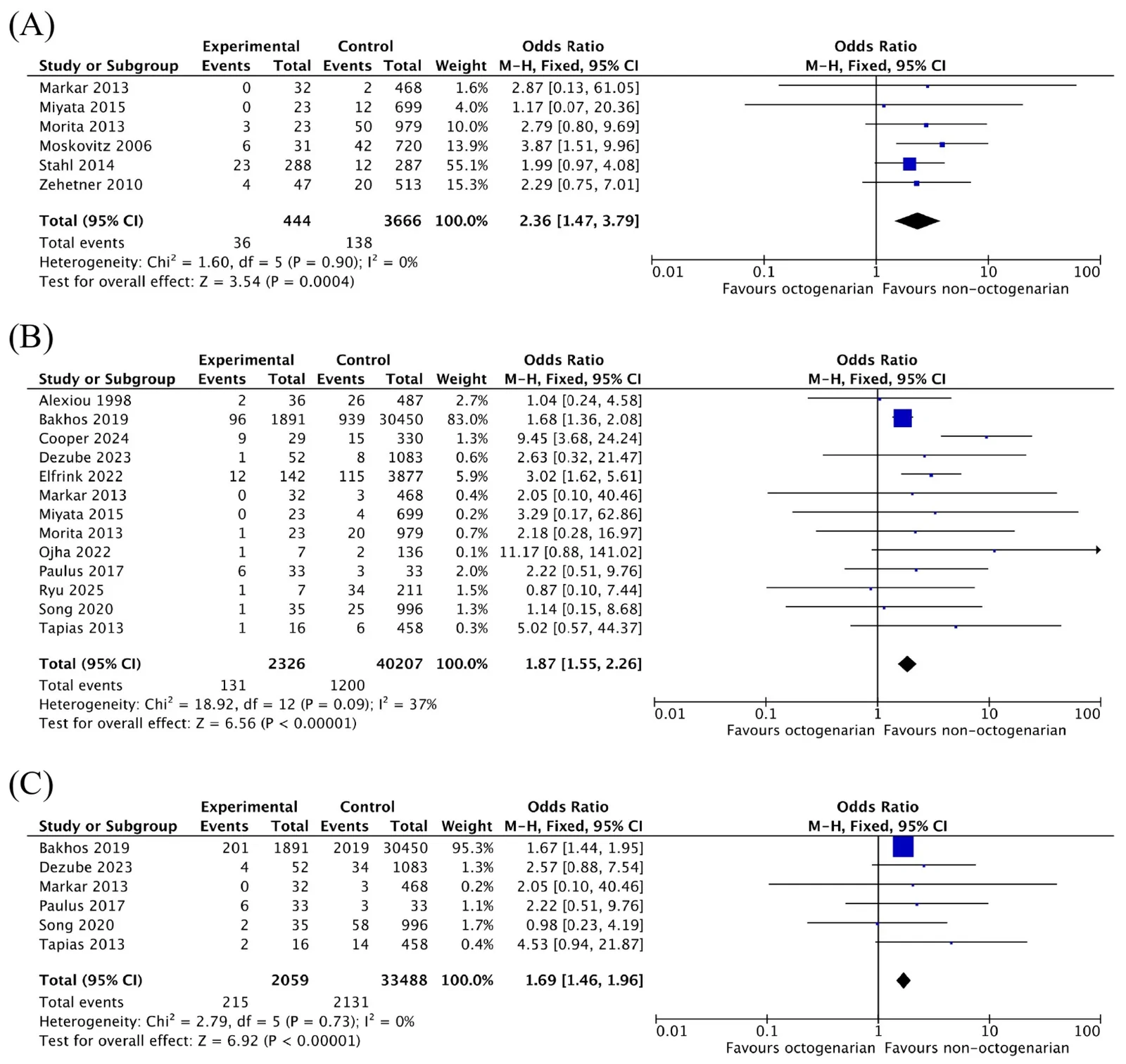
Comparison of (**A**) in-hospital mortality [29,30,31,32,38,40]; (**B**) 30-day mortality [23,24,25,26,27,29,31,34,35,36,37,39] and (**C**) 90-day mortality [24,26,29,35,37,39] between octogenarians and non-octogenarians in the included studies.

Pooled analysis of in-hospital mortality showed a significantly higher incidence in octogenarians (*n* = 36/444, 8.1%) compared with non-octogenarians (*n* = 138/3666, 3.8%) (*n* = 6 studies, 4110 patients; OR 2.36; 95% CI: 1.47–3.79; I^2^ = 0%; *p* = 0.0004) (Figure 4A) [29,30,31,32,38,40]. Of the three studies that reported the causes of mortality in octogenarians (*n* = 13), 30.8% (*n* = 4/13) were due to respiratory complications, 15.4% (*n* = 2/13) were due to sepsis and 23.1% (*n* = 3/13) were due to anastomotic leakage [31,32,40]. The pooled 30-day mortality was also significantly higher in octogenarians (*n* = 131/2326, 5.6%) compared with non-octogenarians (*n* = 1200/40,207, 3.0%) (*n* = 13 studies, 42,533 patients; OR 1.87; 95% CI: 1.55–2.26; I^2^ = 37%; *p* < 0.00001) (Figure 4B) [23,24,25,26,27,29,30,31,34,35,36,37,39]. This trend was similarly observed for 90-day mortality (*n* = 6 studies, 35,547 patients; OR 1.69; 95% CI: 1.46–1.96; I^2^ = 0%; *p* < 0.00001) (Figure 4C) [24,26,29,35,37,39].

There were seven studies that reported median length of hospital stay (LOS) [23,25,26,27,29,32,38]; median LOS was 10–26 days for octogenarians and 9–11 days for non-octogenarians. Only two studies reported readmission rates [26,38], of which the incidence was 18.5% (*n* = 63/340) in octogenarians and 13.5% (*n* = 185/1370) in non-octogenarians. There were three studies that reported revision surgery [26,29,35], of which 12.0% (*n* = 14/117) of patients in the octogenarian group and 15.2% (*n* = 241/1584) of patients in the non-octogenarian group underwent revision surgery. None of the studies reported on patient-reported outcome measures or quality-of-life (QoL) indices.

### 3.7. Sensitivity Analysis

Leave-one-out sensitivity analyses were performed for OS and overall post-operative complications. Exclusion of any single study did not alter the direction or statistical significance of the pooled effect sizes. For OS, the pooled HR ranged from 1.84 to 2.47 following sensitivity analysis. Although exclusion of the study by Song et al. resulted in the largest change in magnitude (pooled HR 1.84 (95% CI: 1.71, 1.97) vs. overall HR 2.29 (95% CI: 1.38, 3.79)), the association remained statistically significant and in the same direction (Supplementary Figure S1) [37]. For overall post-operative complications, the pooled OR was 1.29–1.54 following sensitivity analysis, with confidence intervals remaining above 1, confirming the consistent higher complication risks among octogenarians (Supplementary Figure S2).

For short-term mortality, sensitivity analysis excluding Bakhos et al. (contributing 83% of the weight) still yielded significantly higher 30-day mortality in octogenarians (*n* = 12 studies, 10,192 patients; OR 2.82; 95% CI: 1.90–4.18; I^2^ = 6%; *p* < 0.00001) [24]. Similarly, sensitivity analysis excluding Bakhos et al. (contributing 95% of the weight) showed significantly higher 90-day mortality in octogenarians (*n* = 5 studies, 3206 patients; OR 2.05; 95% CI: 1.07–3.94; I^2^ = 0%; *p* = 0.03) [24]. These findings support the robustness of the results of our meta-analyses.

## 4. Discussion

The global population is facing a silver tsunami, with increasing overall life expectancy. While old age used to be a relative contraindication to surgery, the introduction of pre-operative optimisation, Enhanced Recovery After Surgery (ERAS) and trans-disciplinary peri-operative management has improved post-operative outcomes by reducing surgical stress and preserving homeostasis [41]. This is evident from updated meta-analysis showing comparable post-operative outcomes and disease-specific survival in octogenarians and non-octogenarians, especially for colorectal cancer [42]. However, unlike colorectal surgery, esophagectomy is more extensive and morbid and bears significantly greater post-operative stress. This raises the question of whether similar outcomes can also be achieved for esophagectomy. Our meta-analysis demonstrated worse OS, higher rates of post-operative complications and cardiovascular complications and higher short-term mortality in octogenarians compared with non-octogenarians.

The findings from our meta-analysis are not unexpected, given that older patients are more prone to adverse outcomes. However, these findings contrast with those from other studies, such as in the field of colorectal surgery, and we sought to explore the reasons. Prior to our study, the last meta-analysis comparing outcomes between octogenarians and non-octogenarians was conducted by Han et al. in 2019 (*n* = 9 studies, 4946 patients) [13]. They similarly showed that octogenarians had lower 5-year OS (HR 2.66, 95% CI 1.65–4.28; *p* < 0.001) and higher in-hospital mortality than non-octogenarians (OR 2.00, CI 1.28–3.13; *p* = 0.002). This is likely explained by the higher co-morbidity burden in octogenarians. However, in our study, clinical demographics were comparable between octogenarians and non-octogenarians. Other possible contributing factors that were not assessed in either their meta-analysis or ours include sarcopenia, frailty and nutritional status. While there is a correlation between old age and frailty, these terms are not synonymous. Frailty is defined as a clinically recognisable state of increased vulnerability from ageing-associated decline in reserves and function across multiple physiologic systems, resulting in impaired ability to cope with acute stressors [43]. A study by Goh et al. showed that amongst older patients who underwent emergency laparotomy, frail patients had a threefold higher 90-day mortality rate [44]. A recent study in Asia also developed a risk stratification tool incorporating sarcopenia, American Society of Anaesthesiologists (ASA) score and clinical frailty scale to predict the risk of 1-year mortality following emergency laparotomy in patients aged ≥65 years [45]. Our review serves as an update to existing evidence and identify if these factors were considered in the original articles included. None of the included studies described frailty, sarcopenia, or nutritional status; nevertheless, four of our included studies agreed upon the importance of frailty assessment in patients undergoing esophagectomy [26,28,29,33]. This raises the importance of geriatric oncology and geriatric surgery principles in the peri-operative management of elderly patients undergoing major surgery. Subsequent studies evaluating outcomes in octogenarians should include frailty, sarcopenia, and nutritional status, as these are distinct entities. The worse outcomes shown in both the previous meta-analysis by Han et al. and our current updated meta-analysis, showing higher post-operative complications and mortality in octogenarians, may be confounded by the presence of frailty and sarcopenia [13].

Given the worse short-term and long-term outcomes shown in our meta-analysis, this raises the question of whether octogenarians should even be offered esophagectomy (with curative intent) given their old age. Factors that should be taken into consideration include (1) whether age alone is a decisive factor, or whether should other confounding factors such as co-morbidity burden, frailty, sarcopenia, and nutritional status be considered (as discussed above); (2) the use of neoadjuvant therapy in octogenarians; (3) prehabilitation and the availability of specialised trans-disciplinary services, including a team of geriatricians, oncologists, dieticians and physiotherapists; (4) the role of MIE, especially for older patients; and (5) the importance of involving family members in the peri-operative management of octogenarians.

The use of neoadjuvant therapy is a cornerstone in the management of locally advanced EC. However, older patients who are frail and have multiple co-morbidities may not be suitable candidates for neoadjuvant therapy. Landmark trials in EC, such as the CROSS trial and the NEOCRTEC5010 trial, excluded patients ≥75 years and 70 years, respectively, thereby limiting the generalisability of their results to octogenarians [7,46]. While trials such as ESOPEC included patients up to 86 years old, data on the proportion of octogenarians who successfully completed the full regimen remain unclear [47]. Expectedly, pooled results from our review showed that significantly fewer octogenarians received neoadjuvant therapy, and this is likely to be a strong confounding factor affecting oncological outcomes. Worse survival in octogenarians may be due to the lower adoption of neoadjuvant therapy and toxicities following neoadjuvant therapy. This limited the feasibility of intensive peri-operative chemotherapy in octogenarians, potentially shifting management towards surgery and other modalities, such as immunotherapy. Other factors, including pre-existing co-morbidities, frailty, sarcopenia, and/or malnutrition, may further exclude them from being eligible for neoadjuvant therapy and affect surgical fitness. This highlights the importance of careful patient selection to identify those who may benefit from curative esophagectomy. Nevertheless, our study provides important evidence that post-operative surgical complication rates (i.e., similar anastomotic leak, chylothorax and recurrent laryngeal nerve injury rates) following esophagectomy remain acceptable in octogenarians and are comparable to those in younger patients. To add on, it has been shown that, at five years following esophagectomy, QoL recovers to a level comparable to that of the general population in most patients [48]; however, there are limited studies evaluating QoL in octogenarians following esophagectomy. Given the increase in global life expectancy and the availability of adjunct adjuvant treatment options, our findings support the use of esophagectomy as a reasonable and viable treatment strategy in carefully selected octogenarians.

The retrospective study by Cooper et al. on 201 patients with locally advanced EC showed comparable toxicity rates, overall survival and recurrence-free survival between those aged <70 and ≥70 years [49]. However, another study by Appius et al., which similarly used the CROSS regime, reported worse OS in older patients (≥75 years) than in non-octogenarian patients (<75 years) (median OS 15 months vs. 41 months; HR 2.2; *p* = 0.015) [50]. In contrary, the FLOT4 trial showed that the treatment effect of FLOT (fluorouracil plus leucovorin, oxaliplatin and docetaxel) was consistent across various age groups (<60, 60–69 and ≥70 years) in patients with ≥cT2 and/or nodal positive gastroesophageal junction adenocarcinoma [51]. Based on the above, existing studies evaluating outcomes of patients with neoadjuvant chemotherapy in older patients have shown mixed results. While our review showed inferior OS in octogenarians compared with non-octogenarians, this result should be interpreted carefully, taking into account various factors, including pre-existing co-morbidities, frailty and/or sarcopenia, toxicities with inability to tolerate/complete neoadjuvant and/or adjuvant therapy, and possibly the lower adoption of neoadjuvant therapy. Ideally, validation studies of neoadjuvant therapy extended to octogenarians with risk-adjusted survival analyses, will provide an answer to our question. However, this will be difficult because of the significant toxicities of chemotherapy agents. In such cases, upfront surgery should be considered. The ideal situation would be to conduct validation studies to extend neoadjuvant chemotherapy regimens to octogenarians; however, realistically, these studies will be hard to conduct, given that only 13.9% of octogenarians (*n* = 98/705) received neoadjuvant therapy. While the JCOG9907 trial showed that neoadjuvant chemotherapy was superior to adjuvant chemotherapy for clinical stage 2 or 3 esophageal SCC, this is difficult for most octogenarians [52]. Hence, given that our study established the safety of esophagectomy with acceptable short-term post-operative outcomes in octogenarians, there remains a need to develop new peri-operative systemic regimens, including immunotherapy, to improve long-term survival in octogenarians undergoing esophagectomy.

Standard ERAS protocols should also be applied to octogenarians undergoing esophagectomy, such as prehabilitation and the use of minimally invasive surgical access. Specific to esophagectomy, the ERAS Society Guidelines for Perioperative Care in Esophagectomy in 2019 provided strong recommendations for nutritional intervention pre-operatively and moderate recommendations for prehabilitation programmes [16]. In our review, none of the included studies described whether prehabilitation was offered. Nevertheless, while efforts to prove the benefits of prehabilitation are ongoing, given the potential benefits of prehabilitation, it should be offered to octogenarians, especially in the presence of multiple co-morbidities, frailty, sarcopenia and malnutrition, given the extent of morbidity of esophagectomy. Similarly, MIE has been shown to reduce pulmonary complications and improve short-term and long-term health-related QoL [8,53]. Reduction in pulmonary complications is especially important in older patients given their inherently increased risk of pneumonia due to reduced mucociliary clearance, impaired oropharyngeal reflexes, diminished cough reflex and reduced lung elasticity. However, there are special considerations on the use of MIE for older patients with significant respiratory and cardiac co-morbidities. Some considerations include the physiological changes with pneumoperitoneum and CO_2_ insufflation in frail patients with limited cardiopulmonary reserves. Nevertheless, the MIRO trial showed the superiority of hybrid MIE over OE for esophageal cancer in reducing 30-day major morbidity and pulmonary complications [8]. While it would have been ideal to compare outcomes between MIE and OE in octogenarians in our review, none of the included studies included data on this; therefore, no conclusions can be drawn. It is important to note, however, that MIE be used carefully in older patients with significant respiratory and cardiac co-morbidities.

Lastly, as with any major surgery, it is always important to ensure that patients and family members are actively engaged. Effective pre-operative counselling constitutes an integral part of the overall pre-operative assessment process and has been shown to lower post-operative pain score and improve satisfaction scores [54]. While survival is an important outcome following oncological surgery, QoL is increasingly important [55], especially in the context of octogenarians whose life expectancy is more limited. This includes impairment of QoL from the management of post-operative complications (e.g., the need for repeated procedures such as chest tube insertion for pleural effusion, endoscopic stent placement or negative pressure therapy for anastomotic leakage, or even repeat surgery for revision of anastomosis), as well as long-term gastrointestinal side effects such as dysphagia and reflux. The NCCN guidelines suggest definitive chemoradiotherapy as an alternative to curative esophagectomy for octogenarians with esophageal squamous cell carcinoma. For adenocarcinoma, definitive chemoradiotherapy should only be used in patients who are unfit for surgery or who decline surgery. Long-term QoL differences have been reported to be similar between esophagectomy and chemoradiotherapy [56]. While survival is lower with chemoradiotherapy than with esophagectomy [57], tumour-specific symptoms such as dysphagia and pain may improve within six months of chemoradiotherapy [58]. It is therefore important to provide patient-centric care with discussion on the management options, given the high-morbidity of surgery and the inferior survival in octogenarians compared to non-octogenarians.

Our study has several strengths. Firstly, strict selection criteria were used to select studies that compared outcomes of curative esophagectomy for EC in octogenarians vs. non-octogenarians. We did not include studies with lower age cut-offs, since this could have introduced heterogeneity into our synthesis. Moreover, countries with a high burden of EC are experiencing increasingly ageing populations, with China having an average life expectancy approaching 80 years (77.6 years), and Singapore and Japan having high average life expectancies of 83.9 years and 84.5 years, respectively. Therefore, the growing demographic shift in EC warrants further focus on the octogenarian group specifically. Secondly, our meta-analysis incorporated both large national database studies and single-centre series, thereby enhancing the robustness of our conclusion. Notably, sensitivity analysis yielded consistent results, further supporting the reliability of our findings. Lastly, we used the method described by Tierney et al. to estimate HRs from studies that provided Kaplan–Meier curves without effect sizes [21], thereby avoiding a smaller sample size for OS analysis that would have occurred if those studies had been excluded.

Nevertheless, our study has its limitations. Firstly, although we were interested in studying the potential benefits of MIE over OE in octogenarians, subgroup analysis was not feasible due to a lack of stratified data specifically addressing MIE in older patients. Secondly, some of our included studies were large national database studies that provided limited data on patient demographics, tumour characteristics and operative details. This could have hindered a comprehensive understanding of baseline characteristics across studies and introduced challenges in evaluating potential selection bias among octogenarians undergoing esophagectomy. In addition, although all included studies defined octogenarians as patients aged ≥80 years, some included additional age stratifications (e.g., <79 and 70–79 years), which were merged to form the <80 years group and might have introduced minor heterogeneity. Furthermore, the included studies differed substantially in terms of pre-operative co-morbidities, chemoradiotherapy regimens and surgical techniques due to their retrospective nature, which may also have contributed to variability in outcomes. None of the included studies reported on DFS; therefore, OS was analysed instead. Given the inherently lower life expectancy in octogenarians, the observed difference in OS may have been confounded by non-cancer-related mortality, limiting our ability to attribute the survival differences solely to oncological outcomes. Lastly, the lack of QoL reporting limited assessment of functional recovery following esophagectomy.

## 5. Conclusions

Overall survival in octogenarians undergoing curative esophagectomy is worse than that of non-octogenarians. The incidence of overall post-operative complications, cardiovascular complications and short-term mortality is also higher in octogenarians. These findings may be attributed to co-morbidity burden, frailty, sarcopenia, and/or malnutrition. Nevertheless, rates of post-operative surgical complications were comparable between octogenarians and non-octogenarians. Future studies evaluating the utility of MIE and individualised tailored approaches in octogenarians are warranted, with incorporation of geriatric oncology and geriatric surgery principles, including multimodal-prehabilitation, trans-disciplinary care, and consideration of frailty, sarcopenia and nutritional status during patient selection.

## Figures and Tables

**Table 1:** Summary of effect sizes comparing the demographics, tumour characteristics and esophagectomy details between octogenarians and non-octogenarians.

No	Outcome Variable	No. of Studies	Total Number of Patients, n (Octogenarians/Non-Octogenarians)	No. of Patients (%)		Effect Size, OR (95% CI)/MD (95% CI) *	*p*-Value	I^2^, %	Model Used
				Octogenarians	Non-Octogenarians				
Demographics									
1	Age, years	5	2698 (169/2529)	NA		19.13 (18.20, 20.07) *	**<0.00001**	65	RE
2	Male	15	36,016 (3809/32,207)	2667/3809 (70.0)	25,842/32,207 (80.2)	0.60 (0.56, 0.65)	**<0.00001**	18	FE
3	Hypertension	4	2669 (147/2522)	83/147 (56.5)	1178/2522 (46.7)	1.37 (0.66, 2.86)	0.40	74	RE
4	Diabetes mellitus	7	4665 (237/4428)	30/237 (12.7)	555/4428 (12.5)	1.08 (0.73, 1.60)	0.70	13	FE
5	Cardiovascular disease	6	4191 (221/3970)	69/221 (31.2)	838/3970 (21.1)	1.59 (0.96, 2.60)	0.07	60	RE
6	Respiratory disease	6	4190 (221/3970)	29/221 (13.1)	430/3970 (10.8)	1.13 (0.75, 1.70)	0.55	30	FE
Tumour characteristics									
7	Adenocarcinoma	10	6015 (301/5714)	216/301 (71.8)	3344/5714 (58.5)	1.26 (0.87, 1.84)	0.23	0	FE
8	Stage I-II	11	39,118 (2586/36,532)	1931/2586 (74.7)	21,785/36,532 (59.6)	1.37 (0.75, 2.49)	0.30	95	RE
9	Pathological LN	4	3220 (158/3062)	76/158 (48.1)	1558/3062 (50.9)	0.83 (0.60, 1.15)	0.26	0	FE
Esophagectomy details									
9	Neoadjuvant chemoradiotherapy	12	6858 (705/6153)	98/705 (13.9)	2353/6153 (38.2)	0.40 (0.25, 0.64)	**0.0001**	53	RE
10	MIE	7	5273 (585/4688)	124/585 (21.2)	1379/4688 (29.4)	0.87 (0.68, 1.11)	0.27	0	FE
11	R0 resection	8	35,544 (2064/33480)	1759/2064 (85.2)	30,761/33,480 (91.9)	0.51 (0.45, 0.58)	**<0.00001**	10	FE

* Odds ratios and 95% confidence intervals were used for dichotomous outcomes, while mean differences and 95% confidence intervals were used for continuous outcomes. Values in bold are statistically significant, i.e., *p* < 0.05. CI, confidence interval; FE, fixed-effects; I^2^, heterogeneity; LN, lymph node; MD, mean difference; MIE, minimally invasive esophagectomy; NA, not applicable; OR, odds ratio; RE, random-effects.

**Table 2:** Summary of effect sizes comparing survival, post-operative complications and short-term mortality outcomes between octogenarians and non-octogenarians.

No	Outcome Variable	No. of Studies	Total Number of Patients, n (Octogenarians/Non-Octogenarians)	No. of Patients (%)		Effect Size, OR (95% CI)/HR (95% CI) *	*p*-Value	I^2^, %	Model Used
				Octogenarians	Non-Octogenarians				
Survival									
1	Overall survival (OS)	7	35,441 (2060/33,381)	Median OS: median 18.8 (range 10–61.1) months	Median OS: median 62 (range 29.3–145) months	2.29 (1.38, 3.79) *	**<0.00001**	94	RE
Post-operative complications									
2	Overall complications	11	6515 (333/6182)	196/333 (58.9)	3167/6182 (51.2)	1.40 (1.11, 1.78)	**0.005**	38	FE
3	Major complications (Clavien–Dindo ≥ Grade IIIA)	4	5656 (230/5426)	100/230 (43.5)	1661/5426 (30.6)	1.70 (1.30, 2.22)	**0.0001**	49	FE
4	Anastomotic leakage	8	3814 (225/3589)	21/225 (9.3)	365/3589 (10.2)	0.93 (0.57, 1.51)	0.76	0	FE
5	Chylothorax	4	2219 (107/2112)	3/107 (2.8)	41/2112 (1.9)	1.43 (0.47, 4.32)	0.53	0	FE
6	Respiratory complications	5	4133 (165/3968)	47/165 (28.5)	936/3968 (23.6)	1.25 (0.87, 1.78)	0.22	0	FE
7	Surgical site infection	4	1848 (135/1713)	8/135 (5.9)	113/1713 (6.6)	1.00 (0.47, 2.15)	1.00	0	FE
8	Recurrent laryngeal nerve injury	3	2331 (91/2240)	3/91 (3.3)	151/2240 (6.7)	0.57 (0.19, 1.68)	0.31	0	FE
9	Cardiovascular complications	5	3197 (175/3022)	44/175 (25.1)	448/3022 (14.8)	1.97 (1.34, 2.90)	**0.0006**	0	FE
Short-term mortality									
8	In-hospital mortality	6	4110 (444/3666)	36/444 (8.1)	138/3666 (3.8)	2.36 (1.47, 3.79)	**0.0004**	0	FE
9	30-day mortality	13	42,533 (2326/40,207)	131/2326 (5.6)	1200/40,207 (3.0)	1.87 (1.55, 2.26)	**<0.00001**	37	FE
10	90-day mortality	6	35,547 (2059/33,488)	215/2059 (10.4)	2131/33,488 (6.4)	1.69 (1.46, 1.96)	**<0.00001**	0	FE

* Odds ratios and 95% confidence intervals were used for dichotomous outcomes, while mean differences and 95% confidence intervals were used for continuous outcomes. Values in bold are statistically significant, i.e., *p* < 0.05. CI, confidence interval; FE: fixed-effects; I^2^: heterogeneity; MD: mean difference; NA: not applicable; OR: odds ratio; RE, random-effects.

## Data Availability

No new data were created or analysed in this study.

## Supplementary Materials

The following supporting information can be downloaded at: https://www.mdpi.com/article/10.3390/geriatrics11030067/s1, Methods S1: PRISMA (Preferred Reporting Items for Systematic Reviews and Meta-Analyses) checklist; Methods S2: Details of search strategies used; Methods S3: Newcastle–Ottawa Scale risk of bias assessment for included non-randomised observation studies; Table S1: Study characteristics and patient demographics of included studies; Table S2: Esophageal cancer characteristics and esophagectomy details; Table S3: Post-operative complications, mortality and survival outcomes; Table S4: In-hospital mortality rates and causes; Figure S1: Leave-one-out sensitivity analysis of overall survival; Figure S2: Leave-one-out sensitivity analysis of overall post-operative complications.
