# Supplementary material for: Outcomes of Curative Esophagectomy in Octogenarians vs. Non-Octogenarians with Esophageal Cancer: A Systematic Review and Meta-Analysis
Source: Geriatrics (Basel). 2026 Jun 5;11(3):67. doi: 10.3390/geriatrics11030067 (PMC13299997; doi:10.3390/geriatrics11030067)

Supplementary Materials

Title: Outcomes of Curative Esophagectomy in Octogenarians vs. Non-Octogenarians with Esophageal Cancer: A Systematic Review and Meta-Analysis

Supplementary Methods

Supplementary Methods S1: PRISMA (Preferred Reporting Items for Systematic Reviews and Meta-Analyses) checklist

Section and Topic	Item #	Checklist item	Location where item is reported
**TITLE**			
Title	1	Identify the report as a systematic review.	Page 1
**ABSTRACT**			
Abstract	2	See the PRISMA 2020 for Abstracts checklist.	Page 1
**INTRODUCTION**			
Rationale	3	Describe the rationale for the review in the context of existing knowledge.	Page 2
Objectives	4	Provide an explicit statement of the objective(s) or question(s) the review addresses.	Page 2
**METHODS**			
Eligibility criteria	5	Specify the inclusion and exclusion criteria for the review and how studies were grouped for the syntheses.	Page 3
Information sources	6	Specify all databases, registers, websites, organisations, reference lists and other sources searched or consulted to identify studies. Specify the date when each source was last searched or consulted.	Page 2

Section and Topic	Item #	Checklist item	Location where item is reported
Search strategy	7	Present the full search strategies for all databases, registers and websites, including any filters and limits used.	Supplementary Methods S2
Selection process	8	Specify the methods used to decide whether a study met the inclusion criteria of the review, including how many reviewers screened each record and each report retrieved, whether they worked independently, and if applicable, details of automation tools used in the process.	Page 3
Data collection process	9	Specify the methods used to collect data from reports, including how many reviewers collected data from each report, whether they worked independently, any processes for obtaining or confirming data from study investigators, and if applicable, details of automation tools used in the process.	Page 3
Data items	10a	List and define all outcomes for which data were sought. Specify whether all results that were compatible with each outcome domain in each study were sought (e.g. for all measures, time points, analyses), and if not, the methods used to decide which results to collect.	Page 3
	10b	List and define all other variables for which data were sought (e.g. participant and intervention characteristics, funding sources). Describe any assumptions made about any missing or unclear information.	Page 3
Study risk of bias assessment	11	Specify the methods used to assess risk of bias in the included studies, including details of the tool(s) used, how many reviewers assessed each study and whether they worked independently, and if applicable, details of automation tools used	Page 3

Section and Topic	Item #	Checklist item	Location where item is reported
		in the process.	
Effect measures	12	Specify for each outcome the effect measure(s) (e.g. risk ratio, mean difference) used in the synthesis or presentation of results.	Page 3
Synthesis methods	13a	Describe the processes used to decide which studies were eligible for each synthesis (e.g. tabulating the study intervention characteristics and comparing against the planned groups for each synthesis (item #5)).	Page 3
	13b	Describe any methods required to prepare the data for presentation or synthesis, such as handling of missing summary statistics, or data conversions.	Page 3
	13c	Describe any methods used to tabulate or visually display results of individual studies and syntheses.	Page 3
	13d	Describe any methods used to synthesize results and provide a rationale for the choice(s). If meta-analysis was performed, describe the model(s), method(s) to identify the presence and extent of statistical heterogeneity, and software package(s) used.	Page 3
	13e	Describe any methods used to explore possible causes of heterogeneity among study results (e.g. subgroup analysis, meta-regression).	Page 3
	13f	Describe any sensitivity analyses conducted to assess robustness of the synthesized results.	Page 3

Section and Topic	Item #	Checklist item	Location where item is reported
Reporting bias assessment	14	Describe any methods used to assess risk of bias due to missing results in a synthesis (arising from reporting biases).	N.A.
Certainty assessment	15	Describe any methods used to assess certainty (or confidence) in the body of evidence for an outcome.	N.A.
**RESULTS**			
Study selection	16a	Describe the results of the search and selection process, from the number of records identified in the search to the number of studies included in the review, ideally using a flow diagram.	Page 4
	16b	Cite studies that might appear to meet the inclusion criteria, but which were excluded, and explain why they were excluded.	Figure 1
Study characteristics	17	Cite each included study and present its characteristics.	Page 5
Risk of bias in studies	18	Present assessments of risk of bias for each included study.	Supplementary Methods S3
Results of individual studies	19	For all outcomes, present, for each study: (a) summary statistics for each group (where appropriate) and (b) an effect estimate and its precision (e.g. confidence/credible interval), ideally using structured tables or plots.	Tables 1-2, Supplementary Tables S1-S4

Section and Topic	Item #	Checklist item	Location where item is reported
Results of syntheses	20a	For each synthesis, briefly summarise the characteristics and risk of bias among contributing studies.	Supplementary Tables S1-S4
	20b	Present results of all statistical syntheses conducted. If meta-analysis was done, present for each the summary estimate and its precision (e.g. confidence/credible interval) and measures of statistical heterogeneity. If comparing groups, describe the direction of the effect.	Page 7-11
	20c	Present results of all investigations of possible causes of heterogeneity among study results.	Page 7-11
	20d	Present results of all sensitivity analyses conducted to assess the robustness of the synthesized results.	Page 14
Reporting biases	21	Present assessments of risk of bias due to missing results (arising from reporting biases) for each synthesis assessed.	N.A.
Certainty of evidence	22	Present assessments of certainty (or confidence) in the body of evidence for each outcome assessed.	N.A.
**DISCUSSION**			
Discussion	23a	Provide a general interpretation of the results in the context of other evidence.	Page 11
	23b	Discuss any limitations of the evidence included in the review.	Page 14
	23c	Discuss any limitations of the review processes used.	Page 14

Section and Topic	Item #	Checklist item	Location where item is reported
	23d	Discuss implications of the results for practice, policy, and future research.	Page 14
**OTHER INFORMATION**			
Registration and protocol	24a	Provide registration information for the review, including register name and registration number, or state that the review was not registered.	Page 2
	24b	Indicate where the review protocol can be accessed, or state that a protocol was not prepared.	Page 2
	24c	Describe and explain any amendments to information provided at registration or in the protocol.	N.A.
Support	25	Describe sources of financial or non-financial support for the review, and the role of the funders or sponsors in the review.	Page 15
Competing interests	26	Declare any competing interests of review authors.	Page 15
Availability of data, code and other materials	27	Report which of the following are publicly available and where they can be found: template data collection forms; data extracted from included studies; data used for all analyses; analytic code; any other materials used in the review.	Page 15

Supplementary Methods S2: Details of search strategies used

Database	Number of results	Search strategy
PubMed	883	(“esophagectomy”[MeSH Terms] OR “esophageal surgery”[Title/Abstract] OR “esophageal surgery”[Other Term] OR “esophageal neoplasms”[MeSH Terms] OR “esophageal cancer”[Title/Abstract] OR “esophageal cancer”[Other Term]) AND (“older adults”[Title/Abstract] OR “older adults”[Other Term] OR “octogenarian*”[Title/Abstract] OR “octogenarian*”[Other Term] OR “elderly”[Title/Abstract] OR “elderly”[Other Term] OR “geriatric”[Title/Abstract] OR “geriatric”[Other Term])
Embase	1075	(exp esophagectomy/ OR esophageal surgery.m_titl. OR esophageal surgery.mp. OR exp esophagus tumor/ OR esophageal cancer.m_titl. OR esophageal cancer.mp.) AND (older adults.m_titl. OR older adults.mp. OR octogenarian*.m_titl. OR octogenarian*mp. OR elderly.m_titl. OR elderly.mp. OR geriatric.m_titl. OR geriatric.mp.)
Cochrane Library	145	(MeSH descriptor: [Esophagectomy] explode all trees OR “esophageal surgery”:ti,ab OR “esophageal surgery”:kw OR MeSH descriptor: [Esophageal Neoplasms] explode all trees OR “esophageal cancer”:ti,ab OR “esophageal cancer”:kw) AND (“older adults”:ti,ab OR “older adults”:kw OR “octogenarian*”ti,ab OR “octogenarian*”:kw OR “elderly”:ti,ab OR “elderly”:kw OR “geriatric”:ti,ab OR “geriatric”:kw)
Web of science	1538	(TS=(esophagectomy) OR TS=(esophageal surgery) OR TS=(esophageal neoplasms) OR TS=(esophageal cancer)) AND (TS=(older adults) OR TS=(octogenarian*) OR TS=(elderly) OR TS=(geriatric))

Supplementary Methods S3: Newcastle-Ottawa Scale risk of bias assessment for included non-randomized observational studies

Study identification	Representation of the exposed cohort	Selection of the non-exposed cohort	Ascertainment of exposure	Demonstration that outcome of interest was not present at start	Comparability of cohorts on the basis of the design or analysis	Assessment of outcome	Was follow-up long enough for outcomes to occur	Adequacy of follow-up of cohort	Total quality score*
Alexiou 1998	1	1	1	1	2	1	1	1	9
Bakhos 2019	1	1	1	1	2	1	1	1	9
Cooper 2024	1	1	1	1	2	1	1	1	9
Dezube 2023	1	1	1	1	2	1	1	1	9
Elfrink 2022	1	1	1	1	1	1	1	1	8
Finlayson 2007	1	1	1	1	2	1	1	1	9
Markar 2013	1	1	1	1	2	1	1	1	9
Miyata 2015	1	1	1	1	2	1	1	1	9
Morita 2013	1	1	1	1	2	1	1	1	9
Moskovitz 2006	1	1	1	1	2	1	1	1	9

Motoyama 2022	1	1	1	1	1	1	1	1	8
Ojha 2022	1	1	1	1	1	1	0	1	7
Paulus 2017	1	1	1	1	2	1	1	1	9
Ryu 2025	1	1	1	1	2	1	1	1	9
Song 2020	1	1	1	1	2	1	1	1	9
Stahl 2014	1	1	1	1	2	1	1	1	9
Tapias 2013	1	1	1	1	2	1	1	1	9
Zehetner 2010	1	1	1	1	1	1	1	1	8

[*]Quality score of <3: Low quality of evidence, 3-6: Moderate quality of evidence, ≥7: High quality of evidence

Supplementary Tables

Supplementary Table S1: Study characteristics and patient demographics of included studies

**First author, year**	**Study design**	**Study period**	**Country**	**Sample size, n (octogenarian /non-octogenarian)**	**Age (mean (SD))**	**Gender, male**	**Hypertension**	**Diabetes**	**Cardiovascular disease**	**Respiratory disease**	**ECOG Performance Status**	**Charlson Co-morbidity Index**	**ASA score**
Alexiou 1998	RCS	1987 - 1997	United Kingdom	523 (36/487)	Octogenarian: 81.5 (1.7) Non-octogenarian: 63.8 (9.5)	Octogenarian: 52.8% Non-octogenarian: 68.8%	NR	Octogenarian: 11.1% Non-octogenarian: 4.5%	Octogenarian: 16.6% Non-octogenarian: 27.5%	Octogenarian: 2.8% Non-octogenarian: 9.9%	NR	NR	NR
Bakhos 2019	RCS	2004 - 2014	United States	32341 (1891/30450)	NR	NR	NR	NR	NR	NR	NR	Octogenarian: 73.5% (0), 20.9% (1), 5.6% (2) Non-octogenarian: 73.6% (0), 20.9% (1), 5.5% (2)	NR
Cooper 2024	RCS	2012 - 2021	Israel	359 (29/330)	NR	Octogenarian: 62.1% Non-octogenar	NR	NR	NR	NR	NR	Median (IQR) Octogenarian: 6 (6-7)	Octogenarian: 3.4% (1), 27.6% (2), 65.5% (3)

						ian: 69.4%						Non-octogenarian: 4 (3-6)	Non-octogenarian: 17.2% (1), 49.4% (2), 28.8% (3), 1.8% (4)
Dezube 2023	RCS	2005 - 2020	United States	1135 (52/1083)	Octogenarian: 82.5 (2.1) Non-octogenarian: 63.6 (9.2)	Octogenarian: 88.5% Non-octogenarian: 81.2%	Octogenarian: 76.9% Non-octogenarian: 53.0%	Octogenarian: 17.3% Non-octogenarian: 18.3%	Octogenarian: 25.0% Non-octogenarian: 15.1%	Octogenarian: 7.7% Non-octogenarian: 8.4%	Median (IQR) Octogenarian: 1(1) Non-octogenarian: NR	NR	NR
Elfrink 2022	RCS	2014 - 2018	Netherlands	4019 (142/3877)	NR	NR	NR	NR	NR	NR	NR	NR	NR
Finlayson 2007	RCS	1994 - 2003	United States	27950 (3150/24807)	NR	Octogenarian: 69.2% Non-octogenarian: 79.5%	NR	NR	NR	NR	NR	NR	NR
Markar 2013	RCS	1991 - 2011	United States	500 (32/468)	Octogenarian: 82.6 (2.8) Non-octogenarian 63.0 (10.0)	Octogenarian: 82.7% Non-octogenarian: 78.1%	Octogenarian: 43.8% Non-octogenarian: 26.9%	Octogenarian: 3.1% Non-octogenarian: 5.1%	Octogenarian: 18.8% Non-octogenarian: 15.8%	Octogenarian: 9.4% Non-octogenarian: 7.5%	NR	Mean (SD) Octogenarian: 6.6 (0.9) Non-octogenarian: 4.2 (1.3)	Mean (SD) Octogenarian: 2.8 (0.6) Non-octogenarian: 2.6 (0.5)

Miyata 2015	RCS	2000 - 2012	Japan	722 (23/699)	NR	Octogenarian: 87.0% Non-octogenarian: 87.8%	NR	Octogenarian: 17.4% Non-octogenarian: 9.9%	Octogenarian: 39.1% Non-octogenarian: 28.3%	Octogenarian: 21.7% Non-octogenarian: 8.2%	Octogenarian: 56.5% (0), 43.5% (1) Non-octogenarian: 70.4% (0), 26.6% (1), 4.7% (2-3)	NR	NR
Morita 2013	RCS	1964 - 2011	Japan	1002 (23/979)	NR	Octogenarian: 78.3% Non-octogenarian: 88.2%	NR	NR	NR	NR	NR	NR	NR
Moskovitz 2006	RCS	1996 - 2005	United States	751 (31/720)	NR	Octogenarian: 67.7% Non-octogenarian: 74.6%	NR	Octogenarian: 22.6% Non-octogenarian: 15.1%	Octogenarian: 25.8% Non-octogenarian: 17.2%	Octogenarian: 19.4% Non-octogenarian: 11.9%	NR	NR	NR
Motoyama 2022	RCS	2008 - 2011	Japan	1407 (392/1015)	NR	NR	NR	NR	NR	NR	NR	NR	NR
Ojha 2022	RCS	2012 - 2020	United States	143 (7/136)	NR	Octogenarian: 71.4% Non-octogenarian: 86.0%	NR	NR	NR	NR	NR	NR	NR

Paulus 2017	RCS	2000 - 2012	United States	66 (33/33)	Octogenarian: 83.0 (2.4) Non-octogenarian: 62.0 (9.1)	Octogenarian: 78.8% Non-octogenarian: 90.9%	NR	NR	NR	NR	NR	Octogenarian: 27.3% (2), 42.4% (3), 24.2% (4), 6.1% (5) Non-octogenarian: 27.3% (2), 30.3% (3), 24.2% (4), 18.2% (5)	NR
Ryu 2025	RCS	2008 - 2024	Korea	218 (7/211)	NR	Octogenarian: 100% Non-octogenarian: 91.5%	NR	NR	NR	NR	NR	NR	NR
Song 2020	RCS	1999 - 2017	United States	1031 (35/996)	NR	Octogenarian: 91.4% Non-octogenarian: 93.5%	NR	NR	NR	NR	NR	Octogenarian: 100% (≥2) Non-octogenarian: 6.8% (0), 15.0% (1), 78.2% (≥2)	NR

Stahl 2014	RCS	2009 - 2012	United States	575 (288/287)	NR	Octogenarian: 70.1% Non-octogenarian 72.6%	NR	NR	NR	NR	NR	NR	NR
Tapias 2013	RCS	2002 - 2011	United States	474 (16/458)	Octogenarian: 82.2 (1.6) Non-octogenarian: 62.2 (9.6)	Octogenarian: 62.5% Non-octogenarian: 82.3%	Octogenarian: 23.1% Non-octogenarian: 42.8%	Octogenarian: 0% Non-octogenarian: 13.1%	NR	NR	NR	NR	NR
Zehetner 2010	RCS	1992 - 2007	United States	560 (47/513)	NR	Octogenarian: 78.7% Non-octogenarian: 86.2%	Octogenarian: 53.2% Non-octogenarian: 55.0%	Octogenarian: 10.6% Non-octogenarian: 14.0%	Octogenarian: 57.4% Non-octogenarian: 28.1%	Octogenarian: 21.3% Non-octogenarian: 22.0%	NR	NR	NR

ASA, American Society of Anaesthesiologists; *ECOG,* Eastern Cooperative Oncology Group; *IQR,* interquartile range; *NR,* not reported; *RCS,* retrospective cohort study

Supplementary Table S2: Esophageal cancer characteristics and esophagectomy details

First author, year	Tumour location	Lymph node involvement	Clinical Stage	Histology type	Neoadjuvant chemoradiotherapy	Surgery Approach	Esophagectomy Type	Resection Margin
Alexiou 1998	Octogenarian: 2.8% upper third, 19.4% middle third, 63.9% lower third, 13.9% GEJ<br>Non-octogenarian: 2.7% upper third, 20.9% middle third, 48.0% lower third, 28.3% GEJ	Octogenarian 52.8%<br>Non-octogenarian: 62.2%	Octogenarian: 5.6% I, 38.9% IIa, 11.1% IIb, 44.4% III<br>Non-octogenarian: 5.7% I, 26.9% IIa, 10.3% IIb, 57.1% III	Octogenarian: 69.4% adenocarcinoma, 27.8% squamous cell carcinoma, 2.8% undifferentiated and small cell carcinomas<br>Non-octogenarian: 64.5% adenocarcinoma, 32.0% squamous cell carcinoma, 3.5% undifferentiated and small cell carcinomas	NR	Octogenarian: 100.0% open<br>Non-octogenarian: 100.0% open	Octogenarian: 25.0% Ivor-Lewis, 75.0% thoracoabdominal<br>Non-octogenarian: 5.4% transhiatal, 28.1% Ivor-Lewis, 0.4% McKeown, 65.7% thoracoabdominal	Octogenarian: 88.9% R0, 11.1% R1/R2<br>Non-octogenarian: 87.3% R0, 12.7% R1/R2
Bakhos 2019	NR	NR	Octogenarian: 56.6% I, 26.2% II, 15.3% III, 2.0% IV<br>Non-octogenarian: 30.2% I, 32.1% II, 33.2% III, 4.5% IV	NR	NR	Octogenarian: open + MIE (respective rate NR)<br>Non-octogenarian: open + MIE (respective rate NR)	NR	Octogenarian: 84.6% R0, 15.4% R1/R2<br>Non-octogenarian: 91.8% R0, 8.2% R1/R2
Cooper 2024	Octogenarian: 3.4% upper third, 13.8% middle third,	NR	Octogenarian: 3.4% I, 34.5% II, 62.1% III	Octogenarian: 75.9% adenocarcinoma,	Octogenarian: 61.1% (CROSS / FLOT / Others)	Octogenarian: open + MIE (respective rate NR)	Octogenarian: transhiatal + Ivor-Lewis + McKeown (respective rate NR)	NR

	24.1% lower third, 58.6% GEJ<br>Non-octogenarian: 2.7% upper third, 16.1% middle third, 31.8% lower third, 49.7% GEJ		Non-octogenarian: 12.1% I, 22.1% II, 64.2% III, 1.5% IVa	24.1% squamous cell carcinoma<br>Non-octogenarian: 73.0% adenocarcinoma, 27.0% squamous cell carcinoma	Non-octogenarian: 79.1% (CROSS / FLOT / Others)	Non-octogenarian: open + MIE (respective rate NR)	Non-octogenarian: transhiatal + Ivor-Lewis + McKeown (respective rate NR)	
Dezube 2023	Octogenarian: 1.3% upper third, 10.6% middle third, 88.1% lower third + GEJ<br>Non-octogenarian: 1.3% upper third, 10.6% middle third, 88.2% lower third + GEJ	Octogenarian: 40.4%<br>Non-octogenarian: 47.1%	Octogenarian: 4.8% I, 28.6% II, 59.2% III, 7.1% IV<br>Non-octogenarian: 10.5% I, 20.0% II, 47.6% III, 8.0% IV	Octogenarian: 92.3% adenocarcinoma, 7.7% squamous cell carcinoma<br>Non-octogenarian: 84.9% adenocarcinoma, 13.7% squamous cell carcinoma, 1.3% others	Octogenarian: 65.4%<br>Non-octogenarian: 81.5%	Octogenarian: 26.9% open, 67.3% MIE, 5.8% hybrid<br>Non-octogenarian: 29.4% open, 65.9% MIE, 4.7% hybrid	Octogenarian: 3.9% transhiatal, 59.7% Ivor-Lewis, 34.6% McKeown, 1.9% thoracoabdominal<br>Non-octogenarian: 1.2% transhiatal, 49.7% Ivor-Lewis, 48.3% McKeown, 0.8% thoracoabdominal	NR
Elfrink 2022	NR	NR	NR	NR	NR	NR	NR	NR
Finlayson 2007	NR	NR	NR	NR	NR	NR	NR	NR
Markar 2013	NR	NR	Octogenarian: 25.0% I, 53.1% II, 18.8% III<br>Non-octogenarian: 15.6% I, 39.3% II, 36.3% III, 3.6% IV	NR	Octogenarian: 6.3%<br>Non-octogenarian: 39.7%	NR	Overall: 7.4% transhiatal, 33.4% Ivor-Lewis, 59.0% thoracoabdominal	Octogenarian: 96.9% R0, 3.1% R1/R2<br>Non-octogenarian: 92.9% R0, 7.1% R1/R2

Miyata 2015	Octogenarian: 52.5% middle third, 47.8% lower third Non-octogenarian: 18.2% upper third, 46.1% middle third, 35.8% lower third	NR	Octogenarian: 13.0% I, 26.1% II, 60.9% III Non-octogenarian: 23.3% I, 21.2% II, 41.1% III, 14.4% IV	Octogenarian: 100.0% squamous cell carcinoma Non-octogenarian: 94.3% squamous cell carcinoma, 5.7% others	Octogenarian: 17.4% (5-FU + CDDP + 2Gy for total 40-60Gy of radiation) Non-octogenarian: 14.7% (5-FU + CDDP + 2Gy for total 40-60Gy of radiation)	NR	Octogenarian: 4.3% transhiatal, 69.6% Ivor-Lewis, 17.4% McKeown, 8.7% others Non-octogenarian: 2.1% transhiatal, 47.2% Ivor-Lewis, 48.5% McKeown, 2.1% others	Octogenarian: 87.0% R0, 13.0% R1/R2 Non-octogenarian: 96.6% R0, 3.4% R1/R2
Morita 2013	Octogenarian: 4.3% upper third, 56.5% middle third, 39.1% lower third Non-octogenarian: 13.7% upper third, 61.0% middle third, 25.3% lower third	Octogenarian: 39.1% Non-octogenarian: 48.1%	NR	Overall: 1.2% adenocarcinoma, 96.9% squamous cell carcinoma, 1.9% others	NR	NR	NR	NR
Moskovitz 2006	Octogenarian: 90.3% GEJ Non-octogenarian: 85.7% GEJ	NR	NR	NR	Octogenarian: 19.4% Non-octogenarian: 50.7%	NR	NR	NR
Motoyama 2022	NR	NR	Octogenarian: 19.4% I, 36.5% II, 31.1% III, 10.5% IV Non-octogenarian: 23.8% I, 36.7% II,	NR	Octogenarian: 5.4% Non-octogenarian: 8.1%	Octogenarian: 82.1% open, 17.9% MIE Non-octogenarian: 78.7% open, 21.3% MIE	NR	NR

			30.6% III, 6.2% IV					
Ojha 2022	NR	NR	NR	Octogenarian: 71.4% adenocarcinoma, 14.3% squamous cell carcinoma Non-octogenarian: 83.1% adenocarcinoma, 13.2% squamous cell carcinoma, 2.2% neuroendocrine tumour	Octogenarian: 42.9% Non-octogenarian: 80.2%	Octogenarian: 28.6% open, 71.4% MIE Non-octogenarian: 24.3% open, 75.7% MIE	Octogenarian: 100.0% transhiatal Non-octogenarian: 100.0% transhiatal	Octogenarian: 85.7% R0, 14.3% R1/R2 Non-octogenarian: 84.6% R0, 15.4% R1/R2
Paulus 2017	Octogenarian: 14.3% upper third, 14.3% middle third, 71.4% lower third Non-octogenarian: 15.2% upper third, 48.3% middle third, 36.5% lower third	NR	Octogenarian: 6.1% 0, 18.2% I, 27.3% II, 48.5% III Non-octogenarian: 12.1% 0, 6.1% I, 33.3% II, 45.5% III, 3.0% IV	Octogenarian: 75.8% adenocarcinoma, 15.2% squamous cell carcinoma, nr9.1% others Non-octogenarian: 78.8% adenocarcinoma, 12.1% squamous cell carcinoma, 9.1% others	Octogenarian: 18.2% Non-octogenarian: 9.1%	NR	Octogenarian: 81.8% transhiatal, 6.1% Ivor-Lewis, 12.1% esophagogastrectomy Non-octogenarian: 87.9% transhiatal, 6.1% Ivor-Lewis, 6.1% esophagogastrectomy	Octogenarian: 93.9% R0, 6.1% R1/R2 Non-octogenarian: 97.0% R0, 3.0% R1/R2
Ryu 2025		NR	NR	NR	Octogenarian: 0% (5-FU + CDDP + PTX/CB + 1.8Gy for total 45Gy radiation) Non-octogenarian: 16.1% (5-FU + CDDP + PTX/CB + 1.8Gy for total 45Gy radiation)	NR	Octogenarian: 100.0% Ivor-Lewis Non-octogenarian: 100.0% Ivor-Lewis	Octogenarian: 100.0% R0 Non-octogenarian: 88.2% R0, 11.8% R1/R2

Song 2020	Octogenarian:<br>60.0% lower third, 40.0% GEJ<br>Non-octogenarian:<br>1.5% upper third, 7.0% middle third, 55.5% lower third, 34.5% GEJ, 1.3% proximal gastric	NR	Octogenarian:<br>20.0% I,<br>20.0% IIa,<br>6.7% IIb,<br>50.0% III<br>Non-octogenarian:<br>2.2% 0,<br>13.6% I,<br>21.4% IIa,<br>14.5% IIb,<br>44.0% III,<br>3.0% IV	Octogenarian:<br>85.7% adenocarcinoma, 11.4% squamous cell carcinoma, 2.9% others<br>Non-octogenarian:<br>84.6% adenocarcinoma, 13.1% squamous cell carcinoma, 0.9% adenosquamous carcinoma	NR	Octogenarian:<br>71.4% open,<br>22.9% MIE<br>Non-octogenarian:<br>72.3% open,<br>23.9% MIE	Octogenarian: 28.6% transhiatal, 65.7% Ivor-Lewis, 2.9% others<br>Non-octogenarian:<br>13.0% transhiatal,<br>80.8% Ivor-Lewis,<br>2.3% McKeown,<br>0.1% thoracoabdominal,<br>0.5% others	Octogenarian:<br>91.2% R0,<br>8.8% R1/R2<br>Non-octogenarian:<br>94.6% R0,<br>5.4% R1/R2
Stahl 2014	NR	NR	NR	NR	NR	NR	Octogenarian: 66.7% Ivor-Lewis<br>Non-octogenarian:<br>67.7% Ivor-Lewis	NR
Tapias 2013	NR	NR	Octogenarian:<br>21.4% Ia,<br>14.3% Ib,<br>35.7% IIb,<br>28.6% IIIa<br>Non-octogenarian:<br>7.4% 0,<br>19.9% Ia,<br>10.7% Ib,<br>23.1% IIb,<br>20.7% IIIa,<br>3.7% IIIb,<br>0.9% IIIc,<br>1.1% IV	Octogenarian:<br>87.5% adenocarcinoma, 12.5% squamous cell carcinoma	Octogenarian:<br>18.8% (5-FU/CDDP + total 50Gy radiation)<br>Non-octogenarian:<br>47.8% (FU/CDDP + total 50Gy radiation)	Octogenarian:<br>100.0% open<br>Non-octogenarian:<br>91.7% open,<br>8.3% MIE	Octogenarian: 25.0% transhiatal, 43.8% Ivor-Lewis, McKeown 6.3%, 25.0% thoracoabdominal<br>Non-octogenarian:<br>9.8% transhiatal,<br>53.9% Ivor-Lewis,<br>3.7% McKeown,<br>32.3% thoracoabdominal	NR
Zehetner 2010	NR	Octogenarian:<br>57.4%<br>Non-octogenarian:<br>53.4%	Octogenarian:<br>23.4% I, 23.4 II, 48.9% III,<br>2.1% IV	Octogenarian:<br>100.0% adenocarcinoma<br>Non-octogenarian:	NR	Octogenarian:<br>93.6% open,<br>6.4% MIE<br>Non-octogenarian:	Octogenarian: 78.7% transhiatal<br>Non-octogenarian:<br>35.7% transhiatal	NR

			Non-octogenarian: 27.9% I, 16.2% II, 31.2% III, 3.7% IV	100.0% adenocarcinoma		96.1% open, 3.9% MIE		

CB, carboplatin; *CDDP,* cisplatin; *CROSS,* chemoradiotherapy for oesophageal cancer followed by surgery; *FLOT,* fluorouracil + leucovorin + oxaliplatin + docetaxel; *5-FU,* fluorouracil; *GEJ,* gastroesophageal junction; *MIE,* minimally invasive esophagectomy; *NR,* not reported; *PTX,* paclitaxel

Supplementary Table S3: Post-operative complications, mortality and survival outcomes

First author, year	Overall post-operative complication	Anastomotic leakage	Chylothorax	Respiratory complication	Cardiovascular complication	Readmission	Median length of hospital stay, days	Revision Surgery	30-day mortality	90-day mortality	5-year Survival Rate
Alexiou 1998	Octogenarian: 36.1% Non-octogenarian: 27.5%	Octogenarian: 5.6% Non-octogenarian: 5.5%	Octogenarian: 2.8% Non-octogenarian: 4.1%	Octogenarian: 19.4% Non-octogenarian: 18.9%	Octogenarian: 11.1% Non-octogenarian: 3.7%	NR	Octogenarian: 14 Non-octogenarian: NR	NR	Octogenarian: 5.6% Non-octogenarian: 5.3%	NR	Octogenarian: 19.8% Non-octogenarian: 23.9%
Bakhos 2019	NR	NR	NR	NR	NR	NR	NR	NR	Octogenarian: 5.1% Non-octogenarian: 3.1%	Octogenarian: 10.6% Non-octogenarian: 6.6%	NR
Cooper 2024	Octogenarian: 75.9% Non-octogenarian: 56.7%	NR	NR	NR	NR	NR	Octogenarian: 12 Non-octogenarian: NR	NR	Octogenarian: 31.0% Non-octogenarian 4.5%	NR	NR
Dezube 2023	Octogenarian: 65.4% Non-octogenarian: 58.0%	NR	NR	Octogenarian: 46.2% Non-octogenarian: 32.5%	Octogenarian: 38.5% Non-octogenarian: 21.4%	Octogenarian: 11.5% Non-octogenarian: 12.7%	Octogenarian: 12 Non-octogenarian: NR	Octogenarian: 23.1% Non-octogenarian: 21.0%	Octogenarian: 1.9% Non-octogenarian: 0.7%	Octogenarian: 7.7% Non-octogenarian: 3.1%	NR
Elfrink 2022	NR	NR	NR	NR	NR	NR	Octogenarian: 15 Non-octogenarian: 11	NR	Octogenarian: 8.5% Non-octogenarian: 3.0%	NR	NR
Finlayson 2007	NR	NR	NR	NR	NR	NR	NR	NR	NR	NR	NR
Markar 2013	Octogenarian: 68.8%	Octogenarian: 0%	Octogenarian: 3.1%	NR	NR	NR	Octogenarian: 10	Octogenarian: 3.1%	Octogenarian: 0%	Octogenarian: 0%	NR

	Non-octogenarian: 44.9%	Non-octogenarian: 3.8%	Non-octogenarian: 1.7%				Non-octogenarian: 9	Non-octogenarian: 2.8%	Non-octogenarian: 0.6%	Non-octogenarian: 0.4%	
Miyata 2015	Octogenarian: 47.8% Non-octogenarian: 42.9%	Octogenarian: 8.7% Non-octogenarian: 12.3%	Octogenarian: 4.3% Non-octogenarian: 1.0%	Octogenarian: 17.4% Non-octogenarian: 17.3%	Octogenarian: 17.4% Non-octogenarian: 5.6%	NR	NR	NR	Octogenarian: 0% Non-octogenarian: 0.6%	NR	Octogenarian: 29.3% Non-octogenarian: 50.5%
Morita 2013	Octogenarian: 26.1% Non-octogenarian: 40.2%	NR	NR	Octogenarian: 13.0% Non-octogenarian: 18.0%	NR	NR	NR	NR	Octogenarian: 4.3% Non-octogenarian: 2.0%	NR	Octogenarian: 15.8% Non-octogenarian: 35.0%
Moskovitz 2006	NR	Octogenarian: 22.6% Non-octogenarian: 18.2%	NR	Octogenarian: 29.0% Non-octogenarian: 27.1%	Octogenarian: 25.8% Non-octogenarian: 21.1%	NR	Octogenarian: 26 Non-octogenarian: NR	NR	NR	NR	NR
Motoyama 2022	NR	NR	NR	NR	NR	NR	NR	NR	NR	NR	NR
Ojha 2022	Octogenarian: 85.7% Non-octogenarian: 69.9%	NR	NR	NR	NR	NR	NR	NR	Octogenarian: 14.3% Non-octogenarian: 1.5%	NR	NR
Paulus 2017	Octogenarian: 54.5% Non-octogenarian: 45.5%	Octogenarian: 18.2% Non-octogenarian: 12.1%	NR	NR	Octogenarian: 24.2% Non-octogenarian: 21.25	NR	NR	Octogenarian: 3.0% Non-octogenarian: 3.0%	Octogenarian: 18.2% Non-octogenarian: 9.1%	Octogenarian: 18.2% Non-octogenarian: 9.1%	Octogenarian: 24.2% Non-octogenarian: 42.4%
Ryu 2025	NR	Octogenarian: 14.3% Non-octogenarian: 9.5%	NR	NR	NR	NR	NR	NR	Octogenarian: 14.3% Non-octogenarian: 16.1%	NR	NR
Song 2020	Octogenarian: 71.4%	NR	NR	NR	NR	NR	NR	NR	Octogenarian: 2.8%	Octogenarian: 5.7%	NR

	Non-octogenarian: 64.4%								Non-octogenarian: 2.5%	Non-octogenarian: 5.8%	
Stahl 2014	NR	NR	NR	NR	NR	Octogenarian: 19.8% Non-octogenarian: 16.7%	Octogenarian: 12 Non-octogenarian: 11	NR	NR	NR	NR
Tapias 2013	Octogenarian: 87.5% Non-octogenarian: 55.9%	Octogenarian: 0% Non-octogenarian: 4.8%	Octogenarian: 0% Non-octogenarian: 1.3%	NR	NR	NR	NR	NR	Octogenarian: 6.3% Non-octogenarian: 1.3%	Octogenarian: 12.5% Non-octogenarian: 3.1%	Octogenarian: 49.2% Non-octogenarian: 58.3%
Zehetner 2010	Octogenarian: 53.2% Non-octogenarian: 59.85	Octogenarian: 6.4% Non-octogenarian: 11.1%	NR	NR	NR	NR	NR	NR	NR	NR	NR

NR, not reported

Supplementary Table S4: In-hospital mortality rates and causes

First author, year	In-hospital mortality rate	In-hospital mortality causes in octogenarian group	In-hospital mortality causes in non-octogenarian group
Markar 2013	Octogenarian: 0%<br>Non-octogenarian: 0.4%	NR	NR
Miyata 2015	Octogenarian: 0%<br>Non-octogenarian: 1.7%	NR	1 pneumonia, 2 cardiac insufficiency (remaining causes NR)
Morita 2013	Octogenarian: 13.0%<br>Non-octogenarian: 5.1%	1 stroke, 1 aspiration, 1 cancer death	Respiratory failure, multi-organ failure, pyothorax (specific rate NR)
Moskovitz 2006	Octogenarian: 19.4%<br>Non-octogenarian: 5.8%	3 respiratory complications, 2 anastomotic leak, 1 intraoperative hemorrhage with hypotension and cardiac arrest	NR
Stahl 2014	Octogenarian: 8.0%<br>Non-octogenarian: 4.2%	NR	NR
Zehetner 2010	Octogenarian: 8.5%<br>Non-octogenarian: 3.9%	1 sepsis from small bowel infarction, 1 anastomotic leak with respiratory failure, 1 sepsis from graft ischemia, 1 multiple-organ failure	NR

NR, not reported

Supplementary Figures

Supplementary Figure S1: Leave-one-out sensitivity analysis of overall survival

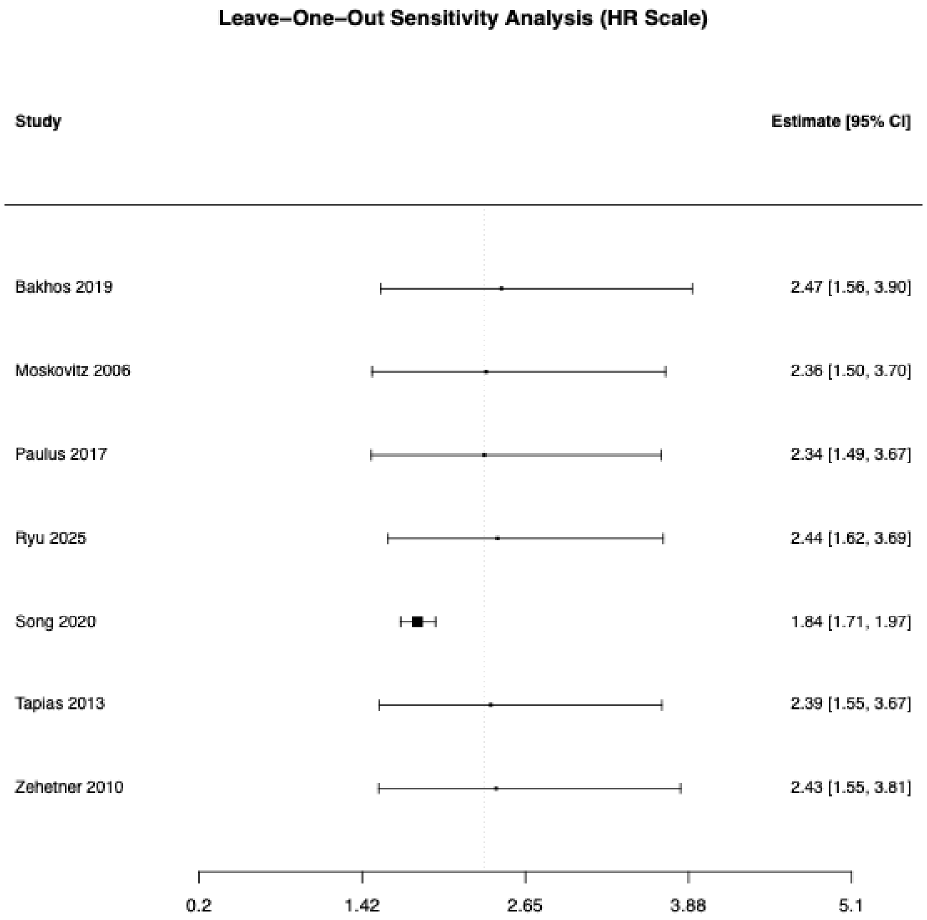

Supplementary Figure S2: Leave-one-out sensitivity analysis of overall post-operative complications

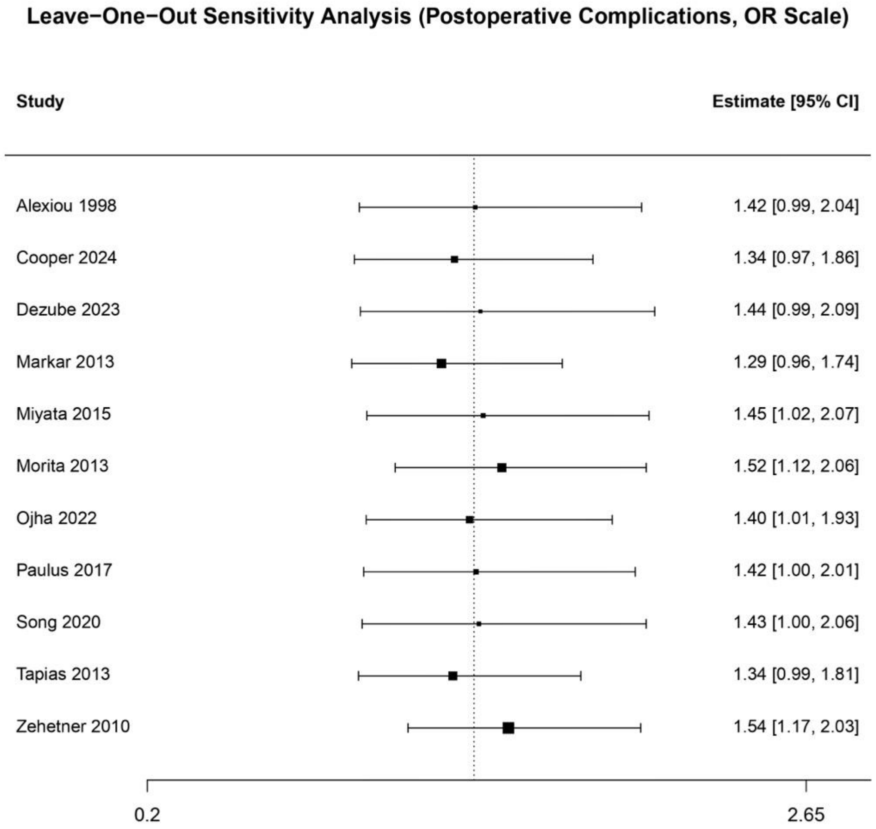

Supplement: Supplementary file 1 [file geriatrics-11-00067-s001.zip › geriatrics-4210930-supplementary.pdf]
